# The Operation of Excision of the Clitoris

**Published:** 1867-08

**Authors:** 


### Miscellaneous.

### The Operation of Excision of the Clitoris.

Mr. Baker Brown's operation—for we believe the senior surgeon of the London Surgical Home has no rival claimant for the questionable honor of recommending the excision of the clitoris for the cure of hysteria, epilepsy, and insanity—has been very properly made the subject of discussion by the Obstetrical Society. It is perhaps a pity that in a question of this kind, which has so many relations to professional ethics as well as to medical science, the Society could not express an authoritative collective opinion on its merits. It is true that the accumulated individual opinions emphatically expressed in condemnation of the *rationale* of the operation, and of the principles which appear to have guided the chief operator in his performance of it, by those who spoke, make up a *quasi*-collective decision that must have great weight. But behind the prominent speakers at a great meeting of a learned Society there is always a large body of men of mature experience, of calm and sagacious judgment, alike free from the fervor of partisanship and proof against the arts of rhetoric. The voice of such a body deliberately given upon the simple question at issue, bared of all complicating and irrelevant incumbrances, would be the best representation of the voice of the profession at large.

But there is another arena for the discussion of this question, which possesses some advantages over a scientific Society. After all, appeal must be made to the whole body of the profession, and that can only be done through the press. The case is now brought to this bar. We cannot shrink from the duty, however repulsive it be, of examining it.

First, then, what is the operation? Secondly, what good is it calculated to effect? The operation has been likened by some to circumcision in the male, but it is more correctly described by Dr. Tyler Smith as analogous to amputation of the penis. Certainly Mr. Brown snips away not only the *præputium clitoridis*, but also the greater part if not the whole of the clitoris itself; and every one must admit that the clitoris is the anatomical homologue of the penis. This is what Mr. Brown, with the pardonable pride of an inventor, means when he speaks of "*my operation*"—his latest if not his greatest discovery. Now the clitoris is undoubtedly a *principal* organ in the large system of erectile and excitable structures in the female. But there are others of scarcely inferior importance; and all are intimately associated to form one whole. The same vascular branches which supply the erectile clitoris supply the other erectile structures adjacent to the ovary, and those which surround the vulva; the pudic nerve, to whose clitoric branches such frightful powers are attributed, also distributes branches to all the other erectile structures of the vulva and vagina. But, contends Mr. Brown—or if he does not so contend then his operation has no meaning—the clitoris is the chief source of peripheral pudic irritation, which, acting on the nervous centres, produces a fearful train of ills, which he thus enumerates:—"1. Hysteria. 2. Spinal irritation, amaurosis, hemiphlegia, etc. 3. Epileptoid fits. 4. Cataleptic fits. 5. Epileptic fits. 6. Idiocy. 7. Mania. 8. Death." Certainly, if this be the true sequence of events, the culmination for which "peripheral irritation of the pudic nerve," or "dilection," as Mr. Brown, in barbarous jargon, otherwise calls it, is held responsible, then Nature was wrong in supplying a clitoris, and the operator of the Surgical Home is right in correcting Nature. But where and how, it will be asked by those deservedly eminent for their knowledge of nervous diseases, has Mr. Brown studied and made this notable discovery? We have lately seen a laudatory paragraph in *The Times*, in which the surgeon of the London Surgical Home is described as having successfully brought insanity within the scope of surgical treatment. Have the physicians of the great lunatic asylums at home and abroad—many of whom are justly celebrated for their profound knowledge of the anatomy, physiology, and diseases of the nervous system, whose lives have been passed in the close observation of men and women suffering from every kind and degree of nervous disease—recognized this sequence? If they have, or shall do, of course they will invite Mr. Brown to make a tour of asylum-deliverance; to hold a grand assize of clitoridectomy. But Mr. Brown does not, so far as we know, cite the evidence of those who are most intimately acquainted with nervous diseases in his favor. He does, indeed, dedicate his book to Brown-Séquard. Does Brown-Séquard endorse Baker Brown? If so, then this repulsive doctrine will be invested with a title to professional respect which it does not as yet possess. Mr. Brown is not, however, so arrogant as to disdain all corroborative testimony. He therefore feels "gratification in being able to name the following gentlemen who have been led to adopt my views and treatment in proper cases:—Sir James Simpson; Beattie, *(sic,)* of Dublin; Sir John Fife and Dr. Dawson, of Newcastle-on-Tyne; Dr. Duke, late of Chichester; Dr. Shettle, of Shaftsbury; John Harrison, Esq., of Chester; Drs. Savage, Routh, and Rogers, in London; my eldest son, Mr. Boyer Brown, now practicing in New South Wales; with my colleagues in the London Surgical Home, Dr. Barratt, and Messrs. Harper, Chambers, I. B. Brown, jr., and Bantock, and very many others."

No doubt these gentlemen will feel it incumbent upon them to relate their own experience and conclusions. Indeed, they stand, cited as they are, in the light of compurgatory witnesses; they cannot, without being liable to misconstruction, maintain silence. Drs. Routh and Rogers have already given their evidence in the discussion at the Obstetrical Society. (See *The Lancet*, February number, p. 119.) Our readers will judge of its value for or against. Still, giving the full measure of weight justly attaching to the names of the gentlemen cited by Mr. Brown, we look for the opinion of others who have had more enlarged opportunities of studying the pathology of nervous diseases. This question of the causation of epilepsy and insanity is of infinitely greater variety and difficulty than Mr. Brown supposes. It is not to be solved by excising the clitoris. It is simply monstrous and contrary to experience to affirm that these diseases are due in any considerable number of instances to unnatural excitation of the pudic nerve. We concur with Dr. West when he says in his admirable letters (see *The Lancet*, February and April numbers,) that "he has not seen any instances in which hysteria, epilepsy, or insanity in woman was *due* to masturbation as its efficient cause." And Dr. Barnes, in his place as President of the Obstetrical Society, declared his conviction that, in the majority of cases of epileptics and insane persons in whom this vicious practice existed, it was resorted to *after* the disease had lasted some time, when the mind had become degraded, and when, being in seclusion, the sexual passion could not be normally gratified.

Has the operation, as a preventive or cure for epilepsy and insanity, a philosophical basis? Certainly this, the first postulate, has not been proved. Can we, accustomed to the rational method of studying medicine, approve the downright empirical method upon which this operation is advocated?

Few physicians will be found to ascribe such dire results to the clitoris. The sources of excitation of the sexual organs are numerous. The periodical congestion of the ovaries occurs independently of the clitoris; the mind alone is sufficient; many accidental conditions of the body produce determinations of blood to the sexual organs, whieh produce the same result; many diseases of the uterus, vagina, and rectum do the same. The prudent physician endeavors to remove or to modify these causes; he does not unnecessarily talk of, or suggest masturbation.

The matter has gone such lengths that it has challenged serious attention, and may possibly call for some decided demonstration on the part of the profession. The question is no longer one simply of the medical or philosophical merits of the operation. It is now surrounded with the most vital questions of moral and professional ethics. We will state one of these, carefully confining ourselves to the published statements of Mr. Brown himself, or of others who accept the responsibility of their allegations. Dr. West says and deliberately repeats (and he is confirmed by Mr. Paget): "I know that this is by no means a solitary instance of the removal of the clitoris by Mr. Brown without the consent, without the knowledge, of the patient." Who will not concur with Dr. West when he says that "the removal of the clitoris without the cognizance of the patient and her friends, without full explanation of the nature of the proceeding, and without the concurrence of some other practitioner selected by the patient or her friends, is in the highest degree improper, and calls for the strongest reprobation?"

This, the moral aspect of the question cannot be evaded. We cannot now pursue it in all its bearings. These deeply concern the honor and public credit of the profession, and must be anxiously examined. Not only is Mr. Brown's operation new, but his views of medical ethics are also new. Are we prepared for a revolution in those principles which, for public good, have governed medical men in the practice of their profession since the days of Hippocrates?—*London Lancet.*

